# The rhizosphere microbiome of burned holm-oak: potential role of the genus *Arthrobacter* in the recovery of burned soils

**DOI:** 10.1038/s41598-017-06112-3

**Published:** 2017-07-20

**Authors:** Antonio J. Fernández-González, Pilar Martínez-Hidalgo, José F. Cobo-Díaz, Pablo J. Villadas, Eustoquio Martínez-Molina, Nicolás Toro, Susannah G. Tringe, Manuel Fernández-López

**Affiliations:** 1 Departamento de Microbiología del Suelo y Sistemas Simbióticos, Estación Experimental del Zaidín, Consejo Superior de Investigaciones Científicas, calle Profesor Albareda 1, 18008 Granada, Spain; 2 Departamento de Microbiología y Genética, Lab. 209. Universidad de Salamanca, edificio Departamental de Biología, Campus M. Unamuno, Salamanca, Spain; 3 DOE Joint Genome Institute, 2800 Mitchell Drive, Walnut Creek, CA 94598 USA

## Abstract

After a forest wildfire, the microbial communities have a transient alteration in their composition. The role of the soil microbial community in the recovery of an ecosystem following such an event remains poorly understood. Thus, it is necessary to understand the plant-microbe interactions that occur in burned soils. By high-throughput sequencing, we identified the main bacterial taxa of burnt holm-oak rhizosphere, then we obtained an isolate collection of the most abundant genus and its growth promoting activities were characterised. 16S rRNA amplicon sequencing showed that the genus *Arthrobacter* comprised more than 21% of the total community. 55 *Arthrobacter* strains were isolated and characterized using RAPDs and sequencing of the almost complete 16S rRNA gene. Our results indicate that isolated *Arthrobacter* strains present a very high genetic diversity, and they could play an important ecological role in interaction with the host plant by enhancing aerial growth. Most of the selected strains exhibited a great ability to degrade organic polymers *in vitro* as well as possibly presenting a direct mechanism for plant growth promotion. All the above data suggests that *Arthrobacter* can be considered as an excellent PGP rhizobacterium that may play an important role in the recovery of burned holm-oak forests.

## Introduction

Wildfires are a recurring threat to the vegetation of the Mediterranean Basin. Moreover, the rural desertion since the 1960s^1^ along with climate change^2^ are causing an increase in forest fires. The effect of wildfires^3, 4^, the recovery of vegetation^5, 6^, the dynamic of microbial communities^7–9^ and nutrient cycling^10, 11^ after forest fires have been given considerable attention. Although understory vegetation could be recovered in a period of time of 20–100 years following a forest fire, microbial communities could recover much faster^9, 12^. However, microorganisms at the recovery stage have never been isolated and characterized on their plant interaction activities.

Microbial communities have a transient alteration in its composition with predominance of spore-forming and Gram-positive microorganisms after a wildfire^7, 8, 10–12^. In this sense, phylum *Actinobacteria* is a unique group of soil microorganisms that now we know are closely associated with plants and they have been isolated from different parts of plants of diverse genera^13^. Several Actinobacteria isolates have been reported to promote plant growth^14–16^. Another important trait, the ability to fix atmospheric nitrogen, has been reported in *Frankia* and other *Actinobacteria* genera: *Arthrobacter* ^17^, *Streptomyces* ^18^ and *Propionibacterium* ^19^. *Arthrobacter* has also been found to degrade a wide variety of compounds, including aromatic molecules, organochloride, pesticides, etc.^20^. In addition, several species of this genus are desiccation tolerant^21, 22^. Recently, it has been described how *Actinobacteria* genera such as *Blastoccocus* and *Arthrobacter* have a main role in the soil nitrogen cycle after a wildfire^11^.

Understanding the main role of the transient dominant microbial population, the new plant-microbe interactions in post-fire conditions as well as the ecological role of these microorganisms is likely to allow a new use as helpers in the recovery of burnt soils and ecosystem. Thus, it is necessary to carry out an adequate phylogenetic analysis based on deep-sequencing of 16S rRNA amplicons from the total prokaryotic communities to reveal the dominant microbial population to isolate them; furthermore “*in vitro*” and “*in planta*” evaluation will allow the correct selection of the microbial strains to be used as plant growth promoting (PGP) rhizobacteria, in order to obtain the desired effect of revegetation of burned soils. For this objective, we use, for the first time, a strategy based on the use of high-throughput technologies for the identification of the key microorganisms found in the tree rhizosphere three years after a wildfire, when the holm-oaks were naturally re-growing. This identification was followed by the isolation of the most abundant genera and their characterization as PGP rhizobacteria to select single isolates that could be used as bacterial inoculants to promote forest recovery after a wildfire.

## Results

### Effects of fire on soil physicochemical properties

Both the control and burned soil samples belong to haplic phaeozem type, classified as loam with regard to their texture (Table S1). Due to the wildfire, there was an increase in soil pH, but in contrast, there was a decrease in soil organic matter and iron concentration. No other shifts due to fire were observed in the physicochemical characteristics of the soil samples assessed (Table S1).

### Bacterial community composition

**Figure 1:**
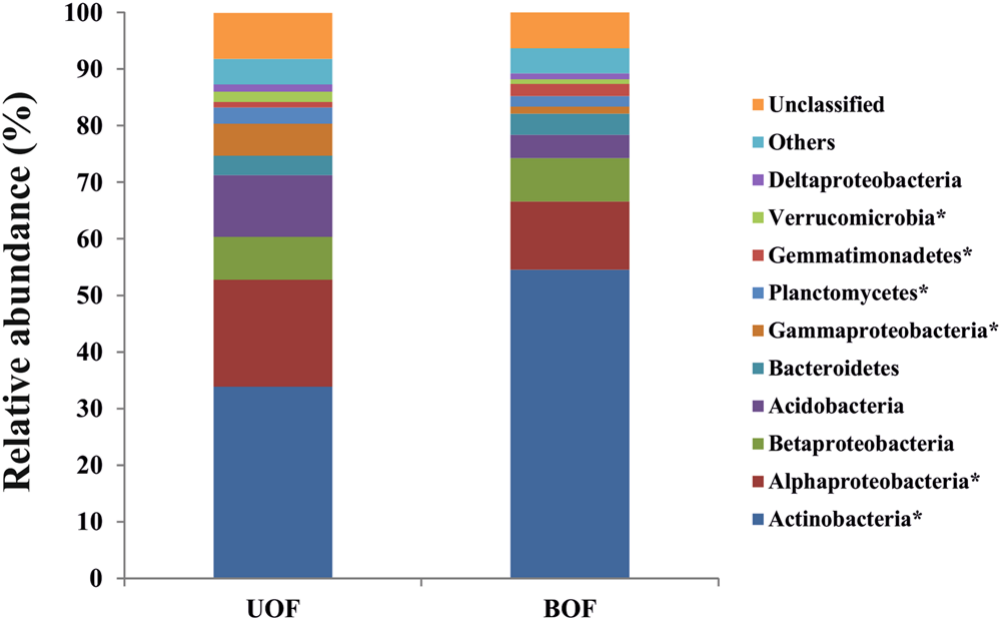
Relative abundances of the dominant bacterial phyla in soils. Relative (%) abundances are based on the proportional frequencies of 16S rRNA gene sequences that could be classified at the phylum level. Asterisks represent a statistically significant difference between UOF and BOF, measured by Fisher’s exact test (p < 0.05). UOF: Undisturbed Oak Forest; BOF: Burned Oak Forest.

Firstly, a total of 62,072 raw reads from BOF (Burned Oak Forest) and 56,620 raw reads from UOF (Undisturbed Oak Forest) were obtained. After the trimming, a total of 22,370 and 14,394 quality sequences were extracted from BOF and UOF, respectively. The sequences were normalized to 4,631 sequences per sample, which was the minimum number at UOF1 sample, thus yielding 13,893 sequences at each rhizospheric situation (Table S2). We were able to classify 92.76% of these sequences. The dominant phyla (or class in the case of *Proteobacteria*) in both samples were *Actinobacteria*, *Alphaproteobacteria*, *Betaproteobacteria*, *Acidobacteria*, *Bacteroidetes*, *Gammaproteobacteria* and *Planctomycetes*, accounting for more than 83% of the bacterial sequences from each of the soils (Fig. 1). In addition, *Gemmatimonadetes*, *Verrucomicrobia* and *Deltaproteobacteria* were present in both soils but at relatively low proportion, and other rare phyla were identified (Table S3).

**Figure 2:**
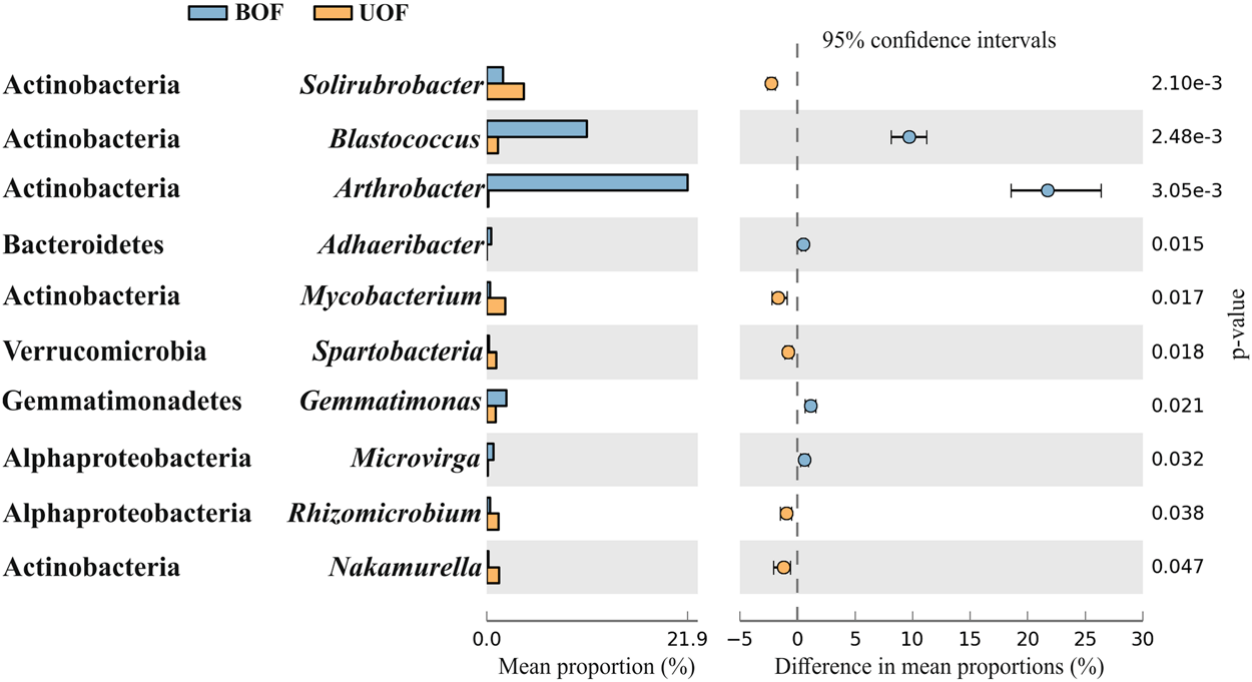
Genera that exhibited significant and biologically relevant changes in abundance. Significant differences were calculated using White’s non-parametric t-test at a 95% confidence interval. Biologic relevance was determined choosing genera with a difference between proportions >0.5 and a ratio of proportions >2. UOF: Undisturbed oak forest; BOF: Burned oak forest.

Fire significantly (p < 0.05) shifted the relative abundance of some dominant phyla (Fig. 1). BOF has more relative abundance of *Actinobacteria* and *Gemmatimonadetes* meanwhile UOF is over-represented in *Alphaproteobacteria*, *Gammaproteobacteria*, *Planctomycetes* and *Verrucomicrobia* the greatest observed differences being in the Gram-positive phyla (Fig. 1). Furthermore, the comparison of the two communities at genus level showed statistically significant differences (p < 0.05) in 26 genera. However, only 10 genera showed biologically relevant differences (Fig. 2). The genera with a significant increase in the BOF rhizosphere were: *Arthrobacter* and *Blastococcus* (phylum *Actinobacteria*), *Adhaeribacter* (phylum *Bacteroidetes*), *Gemmatimonas* (phylum *Gemmatimonadetes*) *and Microvirga* (class *Alphaproteobacteria*). However, other genera from the same phyla were significantly more abundant in the UOF rhizosphere, like: *Solirubrobacter, Mycobacterium* and *Nakamurella* (phylum *Actinobacteria*), *Spartobacteria* (phylum *Verrucomicrobia*) and *Rhizomicrobium* (class *Alphaproteobacteria*).

### Bacterial diversity

In terms of both phylotype (i.e. number of OTUs, Operational Taxonomic Units) and phylogenetic diversity (Table S2), which were analysed at a depth of 13,893 randomly selected sequences per sample, the diversity of bacterial communities was lower in BOF. Phylotype diversity measured with the Shannon index had statistically significant differences, being higher in UOF (6.05) than in BOF (5.07). Good’s coverage was lower in BOF (91.28%) than in UOF (92.50%, Table S2).

### Strains isolation and RAPD fingerprinting

Since the genus *Arthrobacter* had the highest proportion (over 21% of total sequences) and showed a statistically significant difference between BOF and UOF (Fig. 2), we isolated single colonies from rhizospheric BOF soil samples. An average of 100 CFU (Colony-Forming Units) were obtained from 10^−2^ dilution petri dishes, and 231 CFU were selected growing in selective broth media. After filtered selection (see experimental procedures), 55 CFU from the genus *Arthrobacter* were selected.

High-resolution Random Amplification of Polymorphic DNA (RAPD) fingerprints were obtained for the isolated strains (Fig. S1). The amplified fragments ranged from 0.3 to 2.9 kb. We clustered our strains at a 60% similarity level, which defined 12 groups and revealed a high genetic diversity in our isolates. Fifty-one strains were distributed in the clusters containing 2–9 strains per cluster; the remaining 4 isolates had a unique profile. Moreover, no clones were found amongst the isolates.

### Phylogenetic analysis of selected *Arthrobacter* strains

Near complete 16S rRNA gene sequences (1460 bp) were obtained for the 55 selected strains. Sequence similarities between the new isolates and currently described *Arthrobacter* species ranged from 98.99 to 99.93%. A significant number of the isolates sequenced (approx. 62%) showed a 16S rRNA sequence similarity of over 99.5% with the already described *Arthrobacter* species. However, none of them showed a 100% sequence similarity to any type strain in the databases (Table S4).

**Figure 3:**
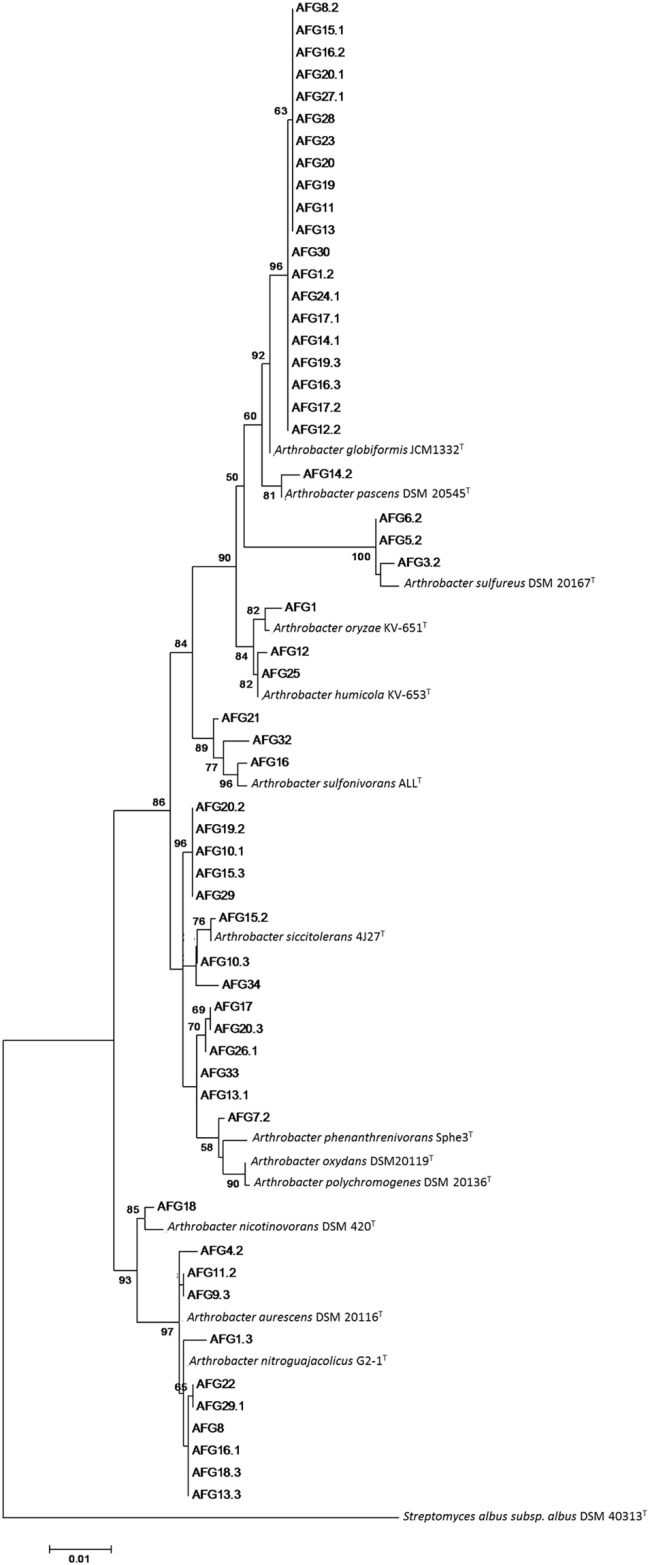
Maximum likelihood phylogenetic tree based on 16S rRNA gene sequences showing the relationship between the 55 isolated *Arthrobacter* strains and the closest recognized *Arthrobacter* species. Bar, 0.01 substitutions per nucleotide position. Bootstrap percentages (1000 replicates) above 50% are shown at nodes.

The inferred phylogenetic tree based on 16S rRNA gene sequences performed using maximum likelihood (Fig. 3) and neighbor-joining methods (Figure S2) showed several groups containing both type strains and our isolates. Other groups contained only sequences from our strains, which indicates the diversity of the isolates from this study compared to the *Arthrobacter* type strains used. A large number of our isolates are closely related to four *Arthrobacter* species: *A. globiformis* (20), *A. siccitolerans* (8), *A. nitroguajacolicus* (7) and *A. oxydans* (5). It should also be noted that the 20 strains related to *A. globiformis* belong to 10 different RAPD groups (Fig. S1) and the same applies to the other strains grouped close to the aforementioned *Arthrobacter* species (Table S4). The tree topology generated by both maximum likelihood and neighbor-joining methods was strongly supported by bootstrap values, which were similar for both methods (Fig. 3 and Fig. S2).

The 16S rRNA gene sequences from the 55 isolates were compared to the pyrotags clustered in the largest OTU from the BOF rhizosphere, all of them with similarity to the *Arthrobacter* 16S rRNA gene. The alignment of the V6-V8 regions from the sequences generated one OTU at 97%, but 46 OTUs at 100% similarity. These 46 OTUs were ordered from major to minor according to the number of pyrotags contained in them (Table 1). The first OTU had 991 pyrotags and it clustered 41 out of 55 sequences from the *Arthrobacter* isolates (Table 1). No isolates were obtained from the second OTU which clustered 468 pyrotags. Three isolates were clustered to the third OTU, which contained 14 pyrotags. The fourth OTU with 7 pyrotags was clustered with 11 isolates (Table 1).

**Table 1:** OTUs clustered at 100% similarity belonging to the genus *Arthrobacter*. Clustering was performed with aligned sequences from 16S rRNA gene region obtained by both pyrosequencing and Sanger method of isolates from rhizospheric BOF soil samples.

OTU	Number of Pyrotags Sequences	Number of Isolates	Species*
1	991	41	*A. globiformis* **20** *A. oxydans* **13** *A. sulfonivorans* **3** *A. humicola* **2** *A. oryzae* **1** *A. pascens* **1** *A. scleromae* **1**
2	468	0	—
3	14	3	*A. sulfureus* **3**
4	7	11	*A. nitroguajacolicus* **10** *A. nicotinovorans* **1**
5	6	0	—
6	6	0	—
7	3	0	—
8	2	0	—
9	2	0	—
10	2	0	—
11	2	0	—

Asterisk indicates species most closely related to the isolates according to near complete 16S rRNA gene BLASTn results, identity ≥ 99%.

### Effect of *Arthrobacter* strains isolated from BOF soil on plant growth

The role of the 55 *Arthrobacter* isolates as putative PGPR was tested in an inoculation experiment with *Medicago sativa* (alfalfa) and *Capsicum annuum* (pepper) plants because plant growth promotion (PGP) experiments on a hardwood like oak are impractical due to time limitations. The reasons for choosing these crops were two-fold. Firstly, we valued a fast confirmation of *in vivo* PGP capabilities of our strains, hence, the use of two crops with a fast growing rate. The second reason for choosing alfalfa and pepper is that these two species are commonly used in such PGP studies and thus are very well characterized. The results on plant growth showed that a large number of *Arthrobacter* strains clearly increased the shoot weight of alfalfa (36, Fig. S3a) or pepper (34, Fig. S3b) and only two of our isolates negatively affected the growth of pepper plants (Fig. S3b). After this preliminary experiment, the best five strains were selected for their ability to significantly increase the weight of the shoot of both inoculated plants (alfalfa and pepper) when compared with control (non-inoculated) plants. Using these five selected strains, a mesocosm experiment was conducted using only alfalfa as the plant of choice (Table 2). The inoculation with any of the five *Arthrobacter* strains selected produced a statistically (p < 0.05) significant higher shoot dry weight than the control treatment. The mean shoot dry weight of plants inoculated with *Arthrobacter* AFG15.2, AFG3.2, AFG16.1, AFG7.2 or AFG20 were, respectively, 53%, 44% 48% 46% and 43% greater than that of control plants (Table 2).

**Table 2:** Growth parameters of alfalfa plants inoculated with selected *Arthrobacte*r strains.

Strain	Length shoot (cm)		Dry weight shoot (mg)	
	Mean	Std. error	Mean	Std. error
Control	34.75	1.75	279.83***	14.73
AFG15.2	38.63	2.32	428.92***	24.24
AFG3.2	37.58	1.66	404.33***	27.26
AFG16.1	38.83	2.24	414.80***	28.71
AFG7.2	39.33	2.24	408.42***	32.29
AFG20	37.29	1.38	400.17**	16.42

Means and standard error (N = 12) are shown. Within columns, treatment means showing significant differences with control plants are marked with asterisks according to Tukey tests at P ≤ 0.1 (*), P ≤ 0.05 (**) and P ≤ 0.01 (***). Uninoculated alfalfa plants were used as control.

### Functional characterization of the selected *Arthrobacter* strains

All of the *Arthrobacter* strains selected showed the ability to grow using Galactose, Glucose and Mannose as the sole carbon source. Furthermore, most of them demonstrated the ability to degrade two or more plant cell wall components, namely cellulose, pectin and xylan (Table 3). Moreover, all of the tested strains showed lipase activity; with them being able to degrade Tween 80, Tween 20 or both. Additionally, all of the isolates showed the ability to produce cellulose with the exception of strains AFG15.2 and AFG7.2 all the *Arthrobacter* strains were able to produce and excrete siderophores. Three isolates (AFG15.2, AFG16.1 and AFG20) were able to solubilize phosphate and only two (AFG16.1 and AFG7.2) were able to grow in N-free medium. Finally, all selected strains were able to produce Indole Acetic Acid (IAA), with the strain AFG7.2 showed production levels at one order of magnitude higher than the rest of the strains (Table 3).

**Table 3:** Ecological, plant growth promoting related enzymatic activities and indolacetic acid production in selected *Arthrobacter* strains.

PPB traits	Cellulase	Pectinase	Xylanase	Tween 20	Tween 80	Cellulose production	Growtn in N free medium	C source Galactose	C source Glucose	C source Manose	C source Xylose	Phosphate solubilization	IAA production	Siderophore production
*Arthrobacter strains*														
AFG 15.2	−	+	+	−	+	+	−	+	+	+	+	+	0.034	−
AFG 3.2	+	−	+	+	+	+	−	+	+	w	−	−	0.034	+
AFG 16.1	−	+	+	+	+	+	+	+	+	+	+	+	0.02	+
AFG 7.2	+	+	+	w	−	+	+	+	+	+	+	−	0.308	−
AFG 20	−	+	+	−	+	+	−	+	+	+	+	+	0.042	+

(+) positive, (−) negative and (w) weak. Indolacetic acid production is expressed in µg ml^−1^.

## Discussion

Three years after wildfire, the physico-chemical analysis of UOF and BOF soils showed minimal differences in mineral composition which should not be relevant for the structure of the microbial community. The main differences between UOF and BOF soils are the increase in pH and the decrease in N and C due to the fire, which according to previous results^11^ are the most important soil factors that influenced the holm-oak rhizospheric communities after wildfire in these sampling sites. The role of pH as an important factor for the structure of the microbial communities is well established^23, 24^. Even after a wildfire, pH had the strongest effect on bacterial community composition and diversity over soil moisture, ammonium content and total nitrogen^9^. In our case, the increase of pH was not an indication of an increase in richness; in fact we report a significant reduction of richness and diversity because of the wildfire (Table S2). This contradiction could be due to the fact that UOF is a slightly acid soil and BOF is a slightly basic soil (Table S1).

The sharpest effect of wildfire in soil microbial community after 3 years was the predominance of phylum *Actinobacteria* mainly due to the high proportion of genus *Arthrobacter* (Figs 1 and 2). The increase of this phylum after the wildfire coincides with the findings of other studies on wildfires by analysis of microbial communities^7, 8, 10^ and metagenomics^11^. It should be pointed out that previous results^11^ were obtained at the same sampling sites but with a metagenomic, functional approach. Thus, our current results obtained by pyrosequencing of 16S rRNA amplicons show that two different molecular approaches offer quite a good comparison, and confirm the main role of the phylum *Actinobacteria* and the genus *Arthrobacter* in microbial communities following Mediterranean oak forest fires. This high proportion of *Actinobacteria* is probably a reflection of its capacity to withstand high temperatures and proliferate on partially sterile burned soils in the form of spores; and in the same way, specific genera like *Arthrobacter* are adapted to oligotrophic conditions. Moreover, the presence of *Arthrobacter* in burned soils could be related to its capacity to form “cyst-like” resting cells and its described physiology to resist starvation, desiccation and oxidative stress^8, 21, 22, 25^.

Therefore, our objective was to obtain and to characterize the dominant bacterial genus from burned soil. Our results showed that the free-living *Arthrobacter* strains isolated in this study were physiologically (Table 3) and genetically diverse (Fig. S1), since a high degree of genetic variation was observed among the 55 isolates when analysed by RAPD fingerprinting. The RAPD grouping provided a useful background for determining the relationship of the isolated strains. It should also be noted that none of them were clones of any other, supporting the idea of the existence of high genetic diversity among isolated *Arthrobacter* strains. The RAPD groups also served to detect strain diversity among those grouped very closely in the phylogenetic tree based on 16S rRNA gene sequences (Fig. 3). RAPD fingerprinting has been shown to be a useful tool to discriminate highly related strains and has been applied to study the genetic diversity of different bacterial taxa^26–28^.

It also should be pointed out that while all our isolates belong to the same genus, they show a very different ability to be cultivated *in vitro* (cultivability, Table 1). With a clustering of OTUs at 100% similarity, we got a ratio of 1 isolate for each 24 sequences of the OTU with highest proportion, which was 1 per each 4 sequences of the third most abundant and not even a single isolate of the second OTU. Others authors have observed a similar discrepancy between cultured microorganisms and the results of clone libraries and pyrosequencing on *Thymus zygis* rhizosphere^29^. The absence of a lineal ratio in the *Arthrobacter* OTUs cultivability could be related to different ecosystemic requirements of each species of the same genus or with the defined composition of the selective media used in this isolation.

Most of the strains in this study have more than 99% 16S rRNA gene similarity with the described *Arthrobacter* species but none of them showed a 100% sequence similarity to any strain type mentioned (Table S4). Despite nearly complete 16S rRNA genes having been sequenced and the high similarity shown, it should not be inferred that our isolates belong to a particular *Arthrobacter* species because of the lack of resolution at the species level of 16S rRNA gene sequence comparisons^30, 31^ (Figs 3 and S2).

The inferred phylogenetic trees based on 16S rRNA gene sequences using maximum likelihood (Fig. 3) and neighbor-joining methods (Fig. S2) were very similar. The topologies of both trees were strongly supported by the high bootstrap values at many branching points which suggest that is very stable (Figs 3 and S2). Taking into account the distribution of our strains in the phylogenetic trees it can be observed that many of these strains are grouped independently from the type strains and could indicate that they were not related to any of the already known *Arthrobacter* species. A more extensive characterization work would be needed to elucidate the taxonomic status of the strains used for this study and the description of new species if needed.

*Arthrobacter* was the bacterial genus that had the highest relative increase in the prokaryotic soil community following the forest fire (Fig. 2). The diversity of *Arthrobacter* strains recovered from the burned soil sample (BOF) (Figs 3, S1 and S2) suggests that members of this genus in the phylum *Actinobacteria* are resistant to drastic changes in environmental conditions associated with wildfire, and could play an important ecological role probably related to the natural recovery of the burned area. One possible ecological role of *Arthrobacter* could be facilitating the recovery of the vegetation cover of the burned areas. *Arthrobacter* spp. can utilize a wide variety of aromatic compounds like those that appear in the soil after a wildfire^32^ and also play an important role in the transformation of organic matter in the natural environment^20^. Moreover, *Arthrobacter* has a main role in the nitrogen cycle of rhizospheric burned soils^11^; therefore, it could play a key role facilitating the change from oligotrophic to copiotrophic conditions. In addition, several *Arthrobacter* species have been described as PGPR and have been used in field revegetation^33–35^, and some of these microorganisms are also tolerant to desiccation^21, 22^.

The role of the 55 *Arthrobacter* isolates as putative PGPR was tested in an inoculation experiment with *Medicago sativa* (alfalfa) and *Capsicum annuum* (pepper) plants. Our goal was to select the microorganisms with PGP activity in both crops. With agronomical characteristics of these plants being very different, positive results in PGP capabilities for both plants would mean that the beneficial effect was not plant-specific. Consequently we could extrapolate that our strains were beneficial to a range of plants, which could include oak. Most of the strains isolated performed well *in planta*, and their PGP effects were significantly different than the control except for two isolates (Fig. S3). This result is very interesting because of its agreement with previous literature in which a high number of beneficial bacteria are systematically found in the rhizosphere^36^, compared to the low numbers of detrimental strains found. This suggests that the plant and/or the bacterial activities favour mutualism. Interestingly in this particular case, the bacterial ecosystem analysed is a burned rhizosphere sample (BOF) and, however, the results are very similar to those described for wild rhizospheric samples. Considering the difficulties of working with oak trees, we decided to scale down the experiments with other plants of interest. *Medicago sativa* is a widely used leguminous plant and *Capsicum annum* has also been stated to have properties that make it a very good model plant for several types of studies such as the study of genetic diversity to develop new cultivars^37^. The PGP results obtained with the two plants are very similar, so we suggest that the observed effect on growth is not dependent on the host and because of the lack of specificity, *Arthrobacter* could help a more rapid recovery of vegetation cover in burned areas, accelerating the growth of plants that would prevent the loss of soil. However, more studies in field conditions in burnt soil would be needed to confirm the role of *Arthrobacter* in vegetation recovery. The five selected strains that were used in the more in-depth study resulted in a 40% increase in growth when compared to the un-inoculated plants. The increase in growth of *M. sativa* in the second experiment (Table 2) is not surprising, given that several studies demonstrate *Actinobacteria*, including strains of *Arthrobacter*, promote both shoot growth in alfalfa as well as in the actinorhizal plant species when co-inoculated with the corresponding nitrogen-fixing micro-symbiont, *Ensifer* or *Frankia*.

As mentioned previously, the increase in shoot growth could be caused by the diverse molecules produced by the *Arthrobacter* strains considered responsible for the PGP action^38, 39^. Siderophore production is commonly found in *Actinobacteria* and is regarded as an important PGPR trait used to overcome low iron availability^40, 41^. The fact that three of our five selected strains produce siderophores is probably related to their capability as PGPR and to the low iron concentration in the BOF sample. Furthermore, IAA production has been directly related with plant growth^42, 43^ and all the *Arthrobacter* strains selected showed IAA production based on the Salkowsky test. Thus, both traits should be considered as general, nonspecific PGP.

Most *Actinobacteria* are saprophytes able to produce a wide range of extracellular hydrolytic enzymes, and all our studied strains synthesize hydrolytic enzymes able to cleave complex polymeric substrates (Table 3), strongly suggesting that *Arthrobacter* can favour plant nutrition by mineralization of soil organic matter. Nonetheless, further research is needed to fully explain the rationale for improved plant growth in plants inoculated with *Arthrobacter*. Phosphorous (P) is also a very limited nutrient which makes the strains able to solubilize phosphate very interesting PGPRs. Three *Arthrobacter* strains showed phosphate solubilisation activity (Table 3), which can thus make soil P more available to plants. Indeed, several *Arthrobacter* isolates have been recently reported as Phosphate-solubilizing rhizobacteria^35, 44^.

The ability to form biofilms plays a fundamental role in the bacterial colonization of the root as well as in the regulation of plant beneficial properties^45^. It has been reported that the production of cellulose is important for biofilm formation^46, 47^. All selected strains are capable of producing cellulose, which will probably aid colonization of plant roots. The ability to fix atmospheric nitrogen is often associated with growth in nitrogen-free media. Our results are preliminary, as it has been suggested before that these methods are not accurate or can lead to falsely positive results^48^ but it is certain that these bacteria can grow in an environment with very limited nitrogen availability, rendering them as excellent candidates for recovering soils, where they could be inoculated to enhance afforestation efforts.

In this study, we obtained and characterized the dominant bacterial genus from a burned rhizospheric soil. Thus, 55 *Arthrobacter* strains were isolated and characterized using RAPDs, 16S rRNA gene sequencing and their interaction when used as an inoculant in pepper and alfalfa plants. It is important to note that *Arthrobacter* is a bacterium that can endure harsh environmental conditions and can naturally be found in soil. But our results indicate that *Arthrobacter* strains isolated from burned soil samples have a very high genetic diversity. We have shown that *Arthrobacter* could play an important ecological role in interacting with the host plant by enhancing aerial growth as a general phenomenon. The selected strains exhibited *in vitro* a great ability to degrade organic polymers as well as possibly presenting a direct mechanism for plant growth promotion (IAA production). It remains to be elucidated whether these positive effects also occur in other plant species. All the above data suggest that, in general, *Arthrobacter* can be considered as an excellent PGPR, although a correct selection of strains is of capital importance because of the detrimental effect that some of them may have for plant growth. However, even though most of the 55 isolates showed a trend towards plant growth enhancement, the five strains examined in detail excel at plant growth promotion. These findings could lead to the formulation of bioinoculants for the recovery of reserves, endangered or endemic plant species.

## Methods

### Experimental site

The study area is located in the Sierra Nevada Natural and National Park (SE Spain); in which, in September 2005, a wildfire burned 3426.74 ha, included 412 ha of evergreen holm oaks (*Quercus ilex* subsp. ballota). Soil samples were collected at the valley of the Lanjarón River, where two sites were selected: one was a holm oak forest affected by the wildfire (burned oak forest, BOF) and another one was a control site in the evergreen oak forest not affected by wildfire (undisturbed oak forest, UOF). These sites are the same sites described previously^11^ and the sampled trees, processes and time of sampling were also the same. The sampling was done three years after the fire, when the holm-oaks were re-growing and a new microbial community, possibly involved in ecosystem recovery, was established. The BOF and UOF sites were on a steep south-facing slope. Three sampling plots were randomly chosen within each study site along transects of 1.0 km length. At the BOF and UOF sites, we sampled the rhizosphere of three trees per plot, each with a diameter of at least 15 cm at breast height and separated by at least 5 m. The specific sampled trees were marked, and the positions of sites were registered with the Global Positioning System (GPS, Fig. S4).

### Sample collection and soil chemical analysis

The rhizospheric samples were collected from a previous work^11^, by following the tree’s main roots until young cork-free roots were found at a distance of less than 50 cm from the trunk. The soil attached to the roots was manually removed and the roots with rhizospheric soil were put into 50 ml Falcon tubes filled with 20 ml of sterilized NaCl 0.8%. After shaking 25 min at 150 rpm and room temperature, the roots were removed and the tubes were centrifuged at 12,850 g for 5 min. The pelleted soil was used to extract environmental DNA. On the other hand, we processed up to 2 kg of soil from each site, sieved through a 2 mm mesh, for physicochemical analysis including soil type, pH, available water, total nitrogen, organic matter, electrical conductivity, etc. All these analyses were carried out with standardized procedures (Table S1) at the Food and Agriculture Laboratory of the Andalusian regional government at Atarfe (Granada, Spain).

### DNA extraction, PCR amplification and pyrosequencing

Soil DNA was extracted from each individual soil sample using the PowerSoil™ DNA Isolation Kit (MoBio, Laboratories Inc., CA), following the manufacturer’s recommendations within 24 hours of sample collection. DNA yields and quality were checked after electrophoresis in 0.8% (w/v) agarose gel stained with ethidium bromide under UV light and with a Nanodrop ND-1000 spectrophotometer (Nanodrop Technologies, Wilmington, DE, USA). An Equimolecular amount of DNA from the three rhizospheres of the same plot was pooled prior to pyrosequencing.

Partial bacterial 16S rRNA gene sequences were obtained from the analysis of each plot sample as described previously^49^ with the amplification of the hypervariable V6-V8 regions with primers 926F (5′-AAACTYAAAKGAATTGRCGG-3′) and 1392R (5′-ACGGGCGGTGTGTRC-3′). The PCR mixtures (25 μl) contained 10 pmol of each primer, 1.8 mM MgCl_2_, 0.4 mM dNTPs, 1 x the corresponding Taq buffer and Taq enhancer buffer, 1 U of Taq Master (5 Prime, USA) and 10 ng of the same DNA template used above. The PCR program consisted of an initial denaturation step at 94 °C for 4 min, 25 cycles of denaturation at 94 °C for 15 s, primer annealing at 55 °C for 45 s and extension at 72 °C for 1 min, followed by a final step of heating at 72 °C for 10 min. For each sample, amplicons were generated in three replicated PCRs. All the amplicons generated from the PCR of each individual sample (9 per treatment) were pooled in equimolar amounts^50–52^ in two composite samples (one composite sample per treatment) that were subjected to pyrosequencing with the Genome Sequencer Titanium FLX system (454| Life Sciences, Branford, CT, USA) at LifeSequencing S.L. (Valencia, Spain). Sequence files were submitted to the NCBI Sequence Read Archive (www.ncbi.nlm.nih.gov/sra) and are available with BioProject accession number PRJNA 291009.

### Pyrosequencing data analysis

Raw sequences were processed using MOTHUR version 1.34.0^53^. Briefly, sequencing errors were reduced using the AmpliconNoise algorithm and low-quality sequences were removed [minimum length of 150 base pairs (bp), allowing 2 mismatch in the barcode, 2 mismatches in the primer, and homopolymers no longer than 8 bp]. Sequences were then aligned using align.seqs function and the SILVA database as template^54^. The chimera.uchime function was then used to identify potentially chimeric sequences which were subsequently removed^55^. Subsequently, the high-quality bacterial sequences were clustered into operational taxonomic units (OTUs) with a similarity of at least 97%. The number of sequences per sample was rarefied before OTU definition. OTU (phylotype richness) distribution among samples was used to calculate rarefaction curves, the phylotype Shannon diversity index (H’), phylotype evenness (Pielou), Chao 1 (abundance-based coverage estimation) richness estimator index, Good’s coverage as well as phylogenetic diversity (phylodiversity)^56^ by the use of MOTHUR software.

Significant differences in Shannon diversity indices between undisturbed and burned samples were assessed using the diversity t test, with p < 0.05 being regarded as statistically significant^57^.

Finally, to examine changes in the relative abundance of the different microbial groups mediated by fire, the normalized bacterial sequences were classified with an 80% bootstrap cutoff to the Ribosomal Database Project (RDP-II) 16S rRNA reference database Release 10^58^ using the classify.seqs function from MOTHUR. Furthermore, significant and biologically relevant differences in the proportions between undisturbed and burned samples at genus level were assessed in STAMP v2.0.9^59^, using White’s non-parametric t-test at a 95% confidence interval. Biologic relevance was determined choosing genera with a difference between proportions >0.5 and a ratio of proportions >2^60^.

### Strains isolation and classification

With the purpose of obtaining cultures of the most abundant genus from burned rhizosphere (BOF), we isolated some bacteria from the first BOF plot (BOF1, located in UTM coordinates, 30S 0458909, 4090493, elevation 1557 m above sea level). From sampling till bacterial isolation, BOF1 soil was stored at −80 °C for three years. The isolation was as follows: Firstly, a pre-enrichment with tryptone 0.5%, 1 g of BOF1 soil and 30 ml dH_2_O (Peptone solution) was gently shaken for one hour at room temperature^61^. Subsequently, after 15 min waiting for the biggest soil particles to decant, 100 μl of Peptone solution was spread over the surface of a sterile HH’ medium in petri dishes and incubated for one week at 30 °C. This process was repeated with serial dilutions up to 10^–7^. The HH’ medium was a modification of a previous medium^62^, and consisted of 0.4% Trypticase soy broth, 0.2% Yeast extract, 3% NaCl, 0.01% Cycloheximide and 1.5% agar.

Following a 7–8 days incubation period, 231 CFU (Colony-Forming Units) were selected. The genomic DNA was obtained by heating 100 μl colony suspensions in dH_2_O at 96 °C for 10 min in a QBD2 dry block heater (Grant, Cambridge, England). This DNA from isolates was amplified with 9bfm forward^63^ and 1512UR reverse^64^ universal primers to almost reach the complete 16S rRNA sequence, with the following PCR specifications: an initial denaturation step at 94 °C for 4 min, 25 cycles of denaturation at 94 °C for 1 min, primer annealing at 52 °C for 1 min and extension at 72 °C for 1.5 min, followed by a final step of heating at 72 °C for 10 min. Partial sequencing of the 16S rRNA gene was performed from forward primer (9bfm) and BLASTed against nt/nr database (https://blast.ncbi.nlm.nih.gov). Finally, 55 CFU from the genus *Arthrobacter* were selected.

### Fingerprinting and phylogenetic analysis of *Arthrobacter* strains

RAPD fingerprinting profiles from bacterial genomic DNA were obtained as described previously^65^. Strain clusters were defined at 60% similarity. The phylogenetic tree was performed once with 55 sequences from 16S rRNA gene from isolates (GenBank accession numbers KT314102-KT314156) and with 63 *Arthrobacter* type strains into RDP-II database. Sequences of reference strains were obtained from the NCBI (http://ncbi.nlm.nih.gov/) and Eztaxon (http://www.ezbiocloud.net/eztaxon) public databases.

For a second tree, *Streptomyces albus* subsp. albus DSM 40313^T^ (GenBank accession number AJ621602.2) was selected as an outgroup and the 12 most closely related type strains were added for the alignment. In addition, the alignment was produced by Infernal Aligner tool^66^ from RDP-II and curated with Geneious R8.1.2. (Biomatters Ltd, Auckland, New Zealand).

Finally, the phylogenetic tree was inferred using the neighbor-joining^67^ and maximum likelihood^68^ methods. Bootstrap analysis was based on 1,000 replicates. MEGA5^69^ was used for neighbor-joining and maximum likelihood analysis.

### Functional characterization of selected *Arthrobacter* strains

Siderophore synthesis was investigated as described previously^70^. Selected strains of *Arthrobacter* were spotted on Chrome Azurol S (CAS) plates; development of deep blue to yellow or orange halo was indicative of siderophore production. Xylan and Pectin were measured as carbon sources using a Yeast Nitrogen Base (Difco) following the manufacturer’s instructions and complemented with xylan or pectin at a 1% concentration. Tween 80 and 20 were added in a concentration of 10 g l^−1^ to a medium containing bacto-peptone (10 g l^−1^), Calcium chloride (5 g l^−1^) and Sodium chloride (0.1 g l^−1^). Degradation halos can be seen in this medium for positive strains.

Cellulose and cellulase production were tested following a procedure stated previously^71^. Degradation of carbon sources was assayed in a TSA medium supplemented with Starch, Gelatin and Casein. Degradation halos appeared in the medium of positive strains.

Phosphate solubilisation activity of bacterial strains was determined by plating onto Pikovskaya’s agar medium^72^ with 0.5% tricalcium phosphate [Ca_3_(PO4)_2_] as the inorganic phosphate source. The plates were incubated at 28 °C for 72 h. The formation of a clear halo around the bacterial colonies indicated phosphate solubilisation.

The indole acetic acid (IAA) production was determined as reported previously^73^. Flasks with GPAM medium supplemented with filter-sterilized L- tryptophan (100 μg ml^−1^) were inoculated with our selected *Arthrobacter* strains (at a concentration of 10^9^ cells per ml) and incubated at 28 ± 2 °C for 4 days. Fully grown cultures were centrifuged at 5,600 g for 5 min. 1 ml of the supernatant was mixed with 1 ml of the Salkowski reagent (50 ml, 35% of perchloric acid, 1 ml 0.5 M FeCl_3_ solution). The mixture was incubated at room temperature for 25 min, and the absorbance of pink colour developed was read at 530 nm by the spectrophotometer Unicam 8625 (Unicam Limited). The concentration of IAA produced by cultures was calculated by using a standard graph of IAA concentrations (range of 10–100 μg ml^−1^).

### Growth in nitrogen-free media

The ability to fix N_2_ was tested on an N-free semisolid SM medium^74^ supplemented with the carbon sources used in the isolation medium. 55 *Arthrobacter* strains were grown in nitrogen-free semisolid agar to determine their potential nitrogen-fixing activity. Strains grown on modified HH’ medium were point inoculated in the bottom of test tubes with 10 ml semi-solid agar, which contained 1% Yeast Carbon Base (Difco, Sparks, MD, USA) and 1% Noble Agar (Difco). The strains were incubated in the dark at 28 °C for 8 days. After the first incubation period, the strains were transferred to fresh media using the first tubes as inoculating source and incubated for an additional 8 days period. The above medium supplemented with (NH_4_)_2_SO_4_ (2 g l^−1^) was used as a positive control.

### Plant assays

*Medicago sativa* var. Aragón seeds were surface-sterilized with HgCl_2_ 2.5% for 5 min and then washed ten times with sterile dH_2_O. Seeds were axenically germinated in petri dishes with filter paper soaked in water for 48 hours. The seedlings were then aseptically transferred to pots with a 1litre capacity, filled with tyndallized agricultural soil. One seedling was aseptically transferred to each pot. *Capsicum annuum* (Pepper) plant experiments were carried out sterilizing the seeds with sodium hypochlorite for 10 min and rinsing them three times in sterile distilled water. Seeds were placed on sterile vermiculite, grown until the first true leaf appeared and then transplanted to pots with commercial substrate. Alfalfa and pepper plantlets were inoculated with 1 ml of bacterial suspensions (10^9^ cells per ml) of each microbial strain. The bacterial suspensions were carefully spotted onto the soil near the roots of the seedling using a micropipette. Ten replicates per treatment were used. Greenhouse conditions were set as follows: 50–60% humidity, with a photoperiod of 16/8 hours. Temperature ranged between 25–15 °C day/night. The plants were harvested 6 weeks after transplantation. The parameters measured were shoot and root dry weight.

In a second experiment, only *Medicago sativa* was used with selected *Arthrobacter* strains. Alfalfa plants were individually grown in pots (1 L volume) containing tyndallized soil in a greenhouse under controlled environmental conditions. The experimental design included 5 treatments with 10 replicates per treatment. Plants were harvested 14 weeks after inoculation.

### Statistical analysis

Plant growth data was studied with univariate (ANOVA) analyses with SPSS v21.0 software for Windows (IBM Corp., Armonk, NY). Dunnett’s one-tailed t-tests were used to identify inoculation treatments with means significantly different from the control at P ≤ 0.05.

## Electronic supplementary material

Supplementary Information
