# Supplementary material for: The rhizosphere microbiome of burned holm-oak: potential role of the genus Arthrobacter in the recovery of burned soils
Source: Sci Rep. 2017 Jul 20;7:6008. doi: 10.1038/s41598-017-06112-3 (PMC5519729; doi:10.1038/s41598-017-06112-3)
Supplement: Supplementary file 1 — Supplementary Information [file 41598_2017_6112_MOESM1_ESM.pdf]

Supplementary Information

The rhizosphere microbiome of burned holm-oak: potential role of the genus *Arthrobacter* in the recovery of burned soils.

Antonio J. Fernández-González, Pilar Martínez-Hidalgo, José F. Cobo-Díaz, Pablo J. Villadas, Eustoquio Martínez-Molina, Nicolás Toro, Susannh G. Tringe, Manuel Fernández-López*

Table S1. Physicochemical properties of sampled soils. EC: Electrical Conductivity. UOF: Undisturbed oak forest; BOF: Burned oak forest.

	Samples	
Soil Variables	UOF	BOF
Clay (%)	21.00	20.50
Sand (%)	45.74	49.54
Silt (%)	33.26	29.96
Textural class	Loam	Loam
pH (H_2O)	6.1	7.6
pH (KCl)	5.7	7.0
Available water (%)	17.11	16.43
EC (mS/cm^3)	0.140	0.220
Organic matter (%)	7.61	4.54
Total N (%)	0.366	0.233
C/N	11.95	11.19
Available P (mg/Kg)	8.0	5.2
K (mg/Kg)	445	330
Na (mg/Kg)	0.065	0.065
Mg (mg/Kg)	4.363	7.203
Ca (%)	12.650	17.972
Fe (mg/Kg)	122	42

Determination of soil parameters.

Texture of soil samples was measured by Bouyoucos hydrometer method (Bouyoucos, 1962). Potentiometric method (Willard et al., 1974; Bates, 1983) was used for the determination of pH. Available water measurement was carried out by gravimetry after drying at a maximum temperature of 105 °C (Gardner, 1986). The method of electrical conductivity was used for determine the salinity. Quantification of total organic carbon was made by volumetric techniques with wet oxidation at controlled temperature (Mebius, 1960). Total nitrogen determination was performed with the Kjeldahl method (Bremner, 1965).Soluble P was measured by Bray method (Bray and Kurtz, 1945), and quantification is performed by colorimetry. Determination exchangeable bases soil was made by spectrometry using ammonium acetate.

Bates, RG. Determination of pH, Wiley, New York. (1983).
Bouyoucos, GS. Hydrometer method improved for making particle size analysis of soils. *Agron. J.* **54**, 464-465 (1962).
Bray, RH and Kurtz, LT. Determination of total, organic and available form of phosphorus in soil. *Soil Sci.* **59**, 360-361 (1945).
Bremner, JM. Inorganic forms of nitrogen. p. 1179-1237. *In* C.A. Black et al. (ed.) Methods of soil analysis. Part 2. Agron. Monogr. 9. ASA, Madison, WI (1965).
Gardner WH. Water content. In Methods of Soil Analysis, Part 1, Klute ed. Am. Soc. Agronomy, Soil Sci. Soc. Am., pp. 493-544 (1986).
Mebius LJ. A rapid method for the determination of organic carbon in soil. *Anal. Chem. Acta* **22**, 120-124 (1960).
Willard HH, Merrit LL, Dean JA. Instrumental methods of analysis. 5th edition Van Nostrand. (1974).

Table S2. Bacterial OTUs phylotype and phylogenetic diversity across samples. Diversity indices were calculated using random selections of 4,631 sequences per soil sample or 13,893 sequences on samples average. Values in parenthesis are 95 % confidence intervals as calculated using MOTHUR. UOF: Undisturbed oak forest; BOF: Burned oak forest. Diversity t test was performed for the burned sample with its control (* significant differences, $p < 0.05$).

	Samples							
Variables	UOF1	UOF2	UOF3	UOF Average	BOF1	BOF2	BOF3	BOF Average
Raw reads	20718	19126	16776	56620	26320	17975	17777	62072
Trimmed reads	4631	5071	4692	14394	10186	6544	5640	22370
Normalized Sequences	4631	4631	4631	13893	4631	4631	4631	13893
OTUs	934	1026	973	977,54±26,58	857	926	866	883±21,53
Chao1	1245 (1170;1343)	1327 (1258;1417)	1192 (1137;1265)	1255±39,27	1377 (1259;1529)	1403 (1298;1537)	1166 (1095;1259)	1315±75,04
InvSimpson	140 (130;152)	200 (185;218)	204 (187;226)	181.33±20.70	20 (19;22)	34 (31;37)	20 (18;21)	24.67±4.67
Shannon	5.89 (5.85;5.94)	6.12 (6.08;6.16)	6.15 (6.11;6.18)	6.05±0.08	4.93 (4.86;5.00)	5.28 (5.22;5.34)	4.99 (4.92;5.06)	5.07±0.11
Pielou	0.86	0.88	0.89	0.88±0.01	0.73	0.77	0.74	0.75±0.01
PhyloDiversity	94.89	99.86	96.88	97.21±1.44	113.76	99.46	84.21	99.14±8.53
Coverage	92.49	91.73	93.29	92.50±0.45	90.98	90.53	92.33	91.28±0.54

Table S3. Relative abundances of phyla classified with RDP-II taxonomy across both soils and the soils separated by different treatments (values represent % of total sequences). UOF: Undisturbed oak forest; BOF: Burned oak forest.

Taxon	Both	UOF	BOF
Actinobacteria	44.19	33.85	54.53
Alphaproteobacteria	15.49	18.90	12.09
Betaproteobacteria	7.62	7.60	7.63
Acidobacteria	7.50	10.88	4.12
Bacteroidetes	3.60	3.45	3.74
Gammaproteobacteria	3.44	5.62	1.25
Planctomycetes	2.36	2.89	1.84
Gemmatimonadetes	1.56	0.99	2.14
Verrucomicrobia	1.31	1.79	0.83
Deltaproteobacteria	1.17	1.30	1.04
Unclassified *Proteobacteria*	1.17	1.73	0.61
Firmicutes	1.15	0.39	1.91
Chloroflexi	0.78	0.93	0.63
Candidate_division_*WPS-2*	0.36	0.30	0.43
Candidate_division_*WPS-1*	0.33	0.32	0.34
Candidatus_*Saccharibacteria*	0.32	0.47	0.17
Armatimonadetes	0.24	0.19	0.29
Chlamydiae	0.06	0.08	0.04
Nitrospirae	0.04	0.04	0.04
Ignavibacteriae	0.03	0.06	0.00
Thaumarchaeota	0.02	0.04	0.00
BRC1	0.01	0.01	0.00
Hydrogenedentes	0.01	0.01	0.00
Unclassified	7.24	6.33	8.15

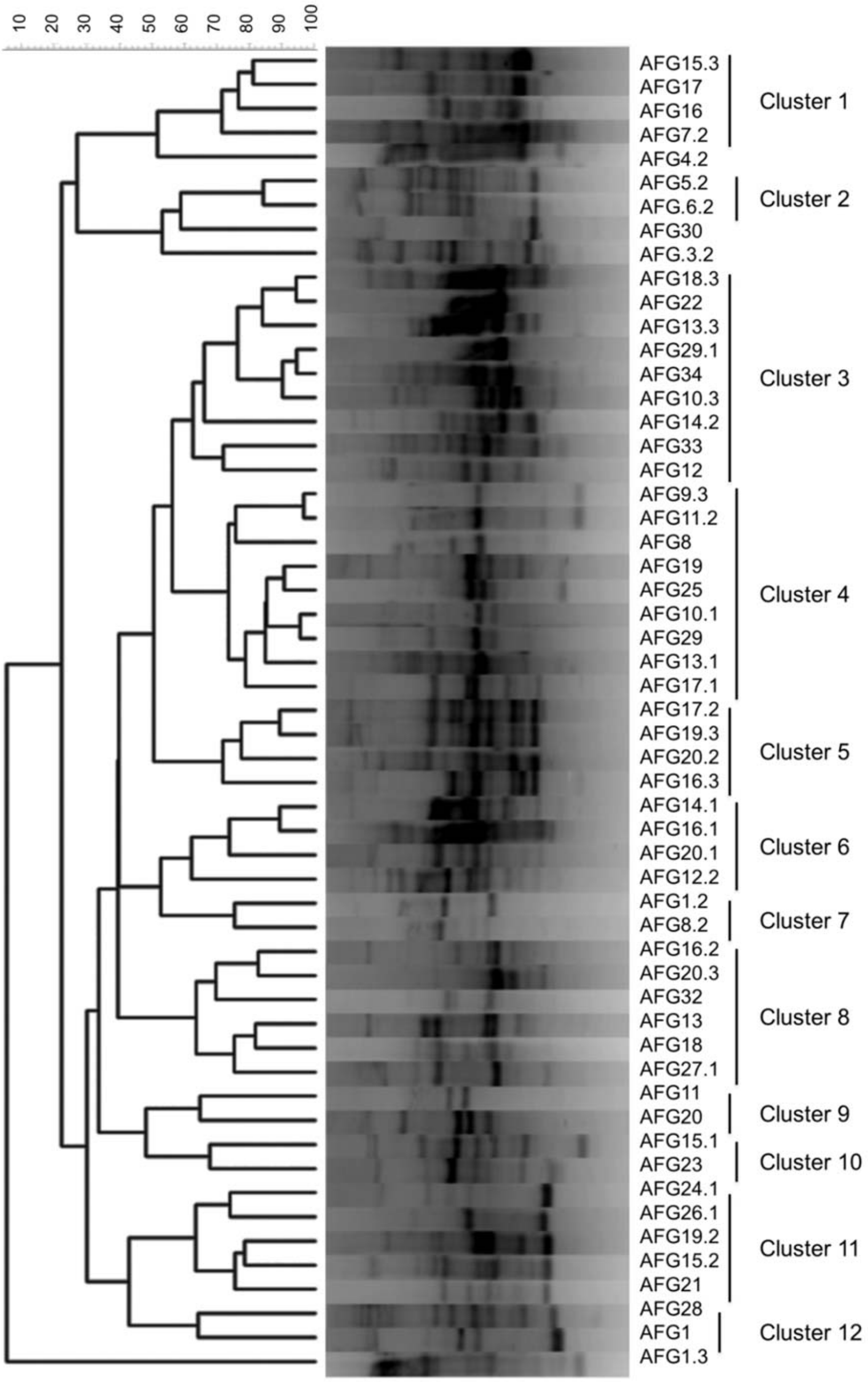

Figure S1. Dendrogram showing genetic relatedness of *55Arthrobacter* strains isolated from burned oak forest soil determined by analysis of RAPD fingerprints using the Pearson's coefficient and UPGMA cluster methods.

Table S4. 16S rDNA gene analysis of strains isolated from burned oak forest (BOF) soil used in this study.

Isolate ID	RAPD group	Accession	Most similar *Arthrobacter* type strain	Accession	Similarity %
AFG4.2	NG	KT314131	*A. aurescens* DSM 20116^T	X83405	99.49
AFG9.3	G-4	KT314144	*A. aurescens* DSM 20116^T	X83405	99.86
AFG11.2	G-4	KT314132	*A. aurescens* DSM 20116^T	X83405	99.86
AFG1.2	G-7	KT314129	*A. globiformis* JCM 1332^T	NR_112192	99.71
AFG8.2	G-7	KT314153	*A. globiformis* JCM 1332^T	NR_112192	99.57
AFG11	G-9	KT314104	*A. globiformis* JCM 1332^T	NR_112192	99.57
AFG12.2	G-6	KT314155	*A. globiformis* JCM 1332^T	NR_112192	99.64
AFG13	G-8	KT314106	*A. globiformis* JCM 1332^T	NR_112192	99.57
AFG14.1	G-6	KT314146	*A. globiformis* JCM 1332^T	NR_112192	99.71
AFG15.1	G-10	KT314156	*A. globiformis* JCM 1332^T	NR_112192	99.57
AFG16.2	G-8	KT314149	*A. globiformis* JCM 1332^T	NR_112192	99.49
AFG16.3	G-5	KT314148	*A. globiformis* JCM 1332^T	NR_112192	99.71
AFG17.1	G-4	KT314145	*A. globiformis* JCM 1332^T	NR_112192	99.71
AFG17.2	G-5	KT314152	*A. globiformis* JCM 1332^T	NR_112192	99.71
AFG19	G-4	KT314110	*A. globiformis* JCM 1332^T	NR_112192	99.49
AFG19.3	G-5	KT314147	*A. globiformis* JCM 1332^T	NR_112192	99.71
AFG20	G-9	KT314111	*A. globiformis* JCM 1332^T	NR_112192	99.57
AFG20.1	G-6	KT314142	*A. globiformis* JCM 1332^T	NR_112192	99.49
AFG23	G-10	KT314114	*A. globiformis* JCM 1332^T	NR_112192	99.57
AFG24.1	G-11	KT314138	*A. globiformis* JCM 1332^T	NR_112192	99.71
AFG27.1	G-8	KT314139	*A. globiformis* JCM 1332^T	NR_112192	99.49
AFG28	G-12	KT314116	*A. globiformis* JCM 1332^T	NR_112192	99.57
AFG30	NG	KT314118	*A. globiformis* JCM 1332^T	NR_112192	99.71

AFG12	G-3	KT314105	*A. humicola* KV-653^T	NR_041546	99.71
AFG25	G-4	KT314115	*A. humicola* KV-653^T	NR_041546	99.93
AFG18	G-8	KT314109	*A. nicotinovorans* DSM 420^T	NR_026194	99.35
AFG1.3	NG	KT314125	*A. nitroguajacolicus* G2-1^T	NR_027199	99.56
AFG8	G-4	KT314103	*A. nitroguajacolicus* G2-1^T	NR_027199	99.93
AFG13.3	G-3	KT314154	*A. nitroguajacolicus* G2-1^T	NR_027199	99.93
AFG16.1	G-6	KT314130	*A. nitroguajacolicus* G2-1^T	NR_027199	99.93
AFG18.3	G-3	KT314140	*A. nitroguajacolicus* G2-1^T	NR_027199	99.93
AFG22	G-3	KT314113	*A. nitroguajacolicus* G2-1^T	NR_027199	99.86
AFG29.1	G-3	KT314141	*A. nitroguajacolicus* G2-1^T	NR_027199	99.86
AFG1	G-12	KT314102	*A. oryzae* KV-651^T	NR_041545	99.64
AFG10.1	G-4	KT314124	*A. oxydans* DSM 20119^T	NR_026236	99.13
AFG15.3	G-1	KT314123	*A. oxydans* DSM 20119^T	NR_026236	99.20
AFG19.2	G-11	KT314143	A. oxydans DSM 20119^T	NR_026236	99.20
AFG20.2	G-5	KT314137	A. oxydans DSM 20119^T	NR_026236	99.20
AFG29	G-4	KT314117	*A. oxydans* DSM 20119^T	NR_026236	99.20
AFG14.2	G-3	KT314151	*A. pascens* DSM 20545^T	NR_026191	99.64
AFG7.2	G-1	KT314127	*A. phenanthrenivorans* Sphe3^T	NR_074770	99.42
AFG10.3	G-3	KT314150	*A. siccitolerans* 4J27^T	NR_108849	99.57
AFG13.1	G-4	KT314136	*A. siccitolerans* 4J27^T	NR_108849	99.64
AFG15.2	G-11	KT314122	*A. siccitolerans* 4J27^T	NR_108849	99.93
AFG17	G-1	KT314108	*A. siccitolerans* 4J27^T	NR_108849	99.49
AFG20.3	G-8	KT314134	*A. siccitolerans* 4J27^T	NR_108849	99.49
AFG26.1	G-11	KT314133	*A. siccitolerans* 4J27^T	NR_108849	99.57
AFG33	G-3	KT314120	*A. siccitolerans* 4J27^T	NR_108849	99.64
AFG34	G-3	KT314121	*A. siccitolerans* 4J27^T	NR_108849	99.13
AFG16	G-1	KT314107	*A. sulfonivorans* ALL^T	NR_025084	99.49

AFG21	G-11	KT314112	*A. sulfonivorans* ALL^T	NR_025084	98.99
AFG32	G-8	KT314119	*A. sulfonivorans* ALL^T	NR_025084	99.06
AFG3.2	NG	KT314126	*A. sulfureus* DSM 20167^T	NR_026237	99.28
AFG5.2	G-2	KT314128	*A. sulfureus* DSM 20167^T	NR_026237	99.49
AFG6.2	G-2	KT314135	*A. sulfureus* DSM 20167^T	NR_026237	99.49

16S rRNA gene sequence analysis: Alignment was performed using nucleotide BLAST algorithm against strain types from nr/nt database. Accession: refers to GenBank accession numbers. Similarity %: shows the percentage of identity to the best BLAST hit with 100 % alignment coverage to each isolate.

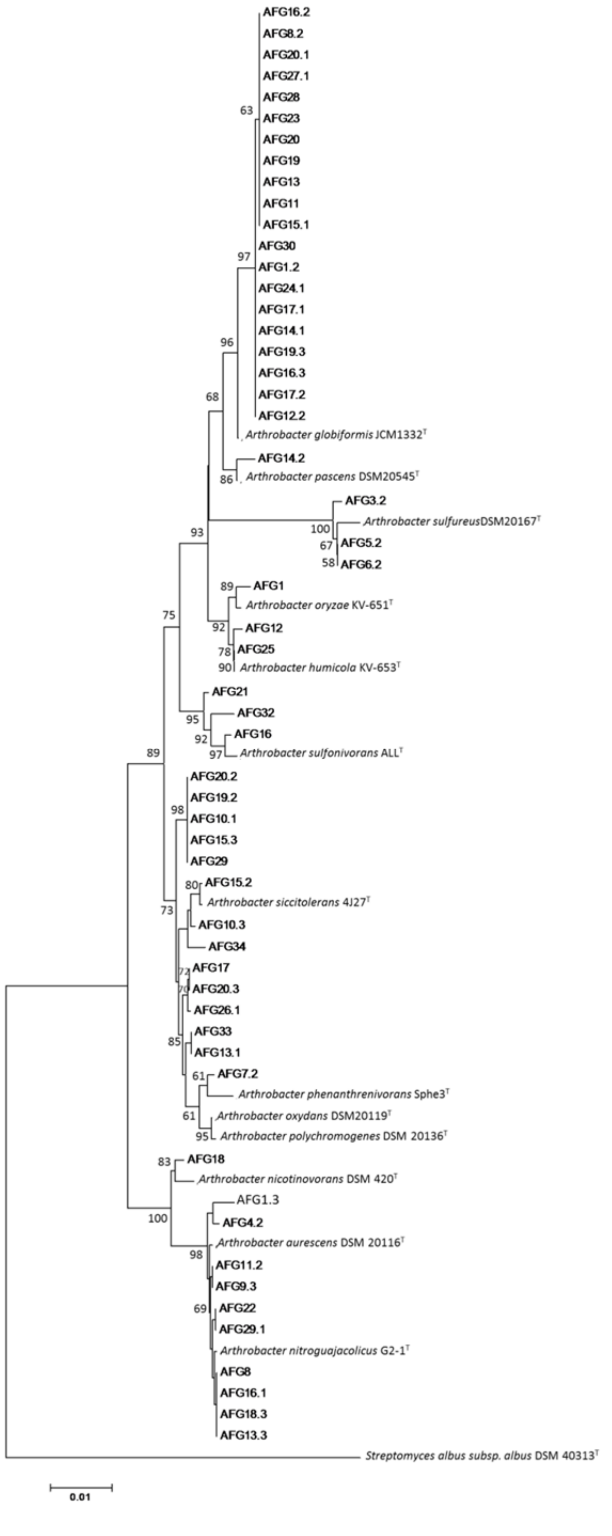

Figure S2. Neighbor-joining phylogenetic tree based on 16S rRNA gene sequences showing the relationship between the 55 isolated *Arthrobacter* strains and the closest recognized *Arthrobacter* species. Bar, 0.01 substitutions per nucleotide position. Bootstrap percentages (1000 replicates) above 50 % are shown at nodes.

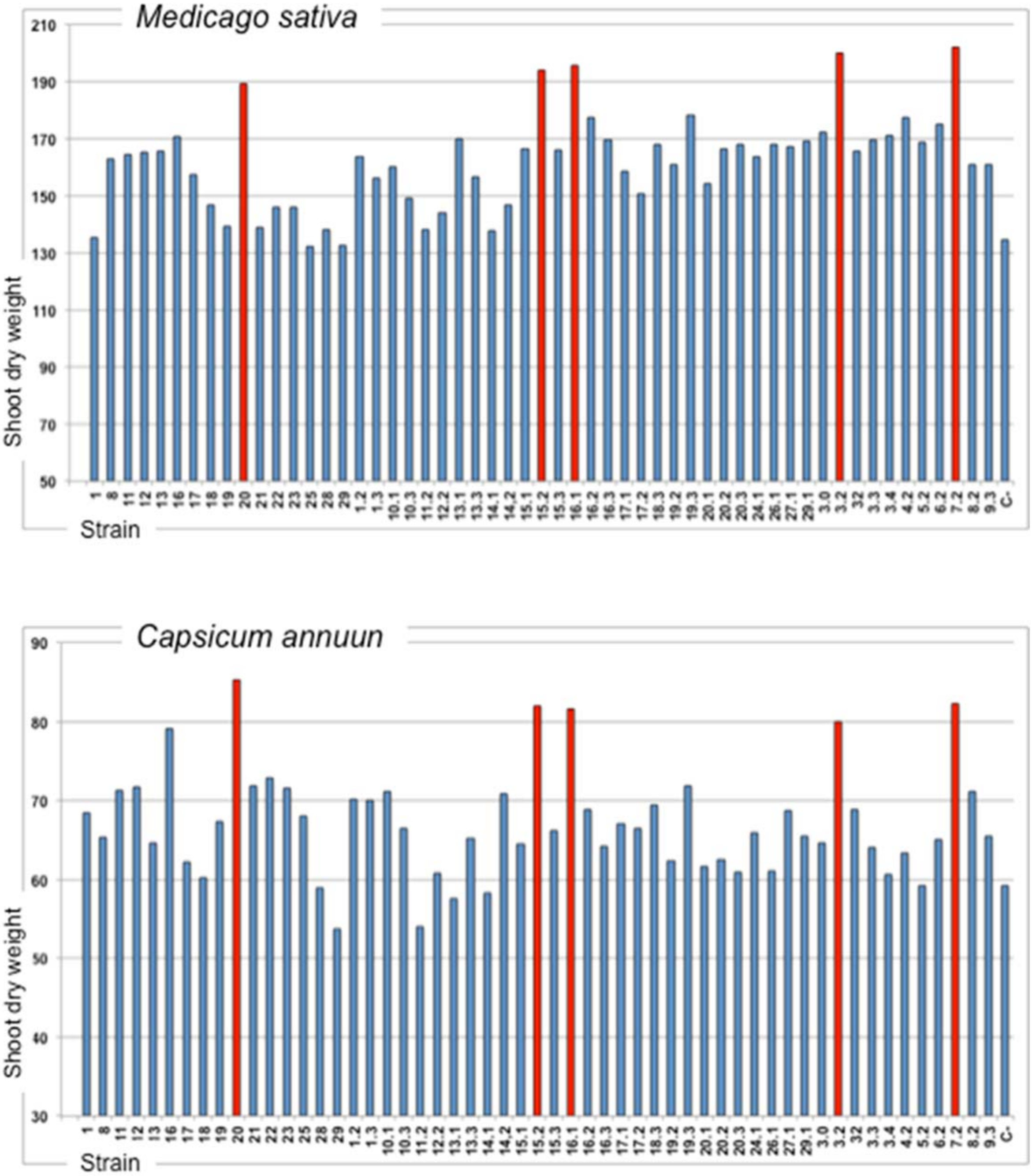

Figure S3. Means shoot dry weight of plants inoculated whit the 55 *Arthrobacter* strains isolated from BOF soil. In red those *Artrhobacter* strains that showed significant (P<0.05) differences respect to the uninoculated controls when inoculated in alfalfa and pepper. Dry weights are expressed in mg.

A)

Site	Altitude	Orientation	Type of area	Geographic coordinates
**UOF**	1790 m asl	South	Undisturbed holm-oak forest	N 36º58’11’’, W 3º27’37’’
**BOF**	1566 m asl	South	Burned holm-oak forest, resprout after wildfire	N 36º57’26’’, W 3º27’48’’

B)

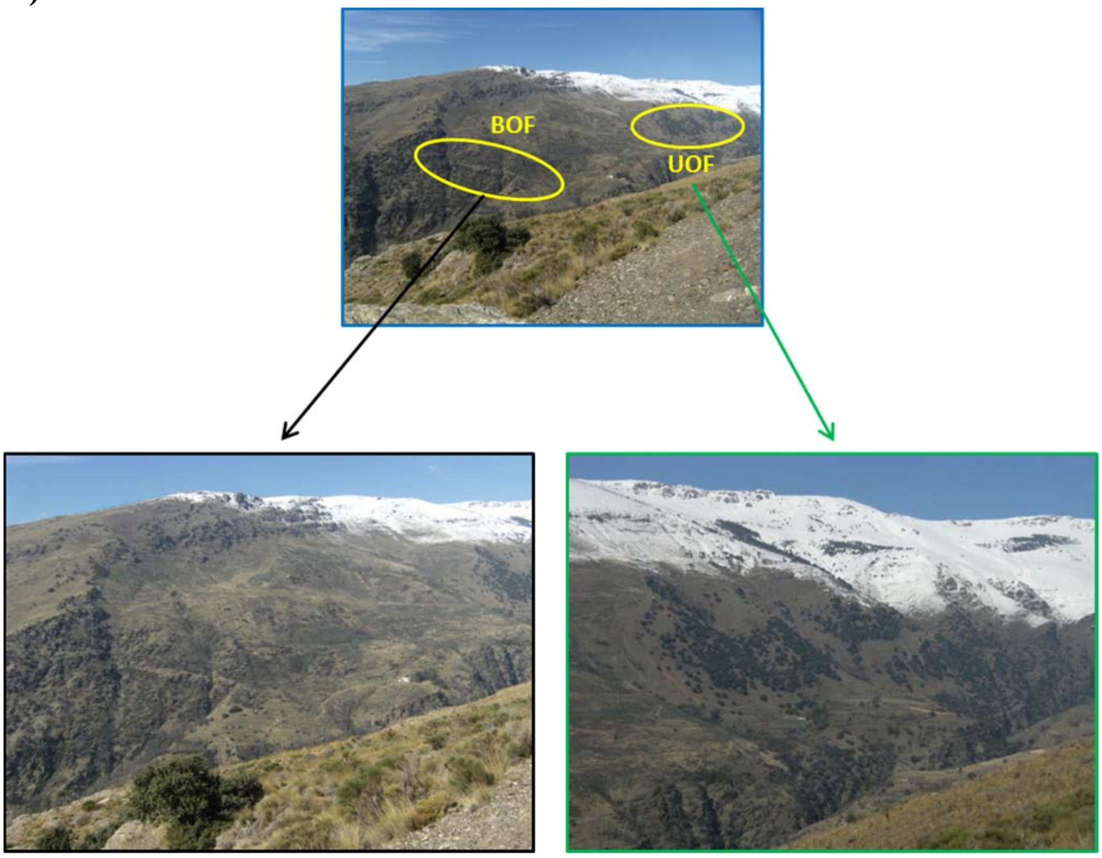

Figure S4. Description of the sampled areas. A) Location of study areas with its geographical coordinates, altitude and orientation. B) Situation of the sampled sites at Sierra Nevada national Park. UOF, undisturbed holm-oak forest; BOF, burned holm-oak forest. Photographs taken by M. Fernandez-Lopez on November 7th, 2008.
